# Role of Cardiovascular Magnetic Resonance in Post-Heart Transplant Surveillance: Integrating Evidence with Prospective Cohort Data

**DOI:** 10.3390/jpm16040201

**Published:** 2026-04-03

**Authors:** Ricardo Carvalheiro, Vera Vaz Ferreira, Ana Raquel Santos, Isabel Cardoso, António Valentim Gonçalves, Rita Ilhão Moreira, Tiago Pereira da Silva, Sílvia Aguiar Rosa, Rui Cruz Ferreira

**Affiliations:** Department of Cardiology, Unidade Local de São José, Hospital de Santa Marta, 1169-024, Lisbon, Portugal

**Keywords:** heart transplantation, cardiovascular magnetic resonance, acute cellular rejection, T2 mapping, multiparametric imaging, graft surveillance, personalized medicine, endomyocardial biopsy

## Abstract

**Background:** Heart transplantation remains the definitive therapy for selected patients with end-stage heart failure, but outcomes are limited by acute rejection, chronic allograft injury, and cardiac allograft vasculopathy. Endomyocardial biopsy (EMB) remains the reference standard for rejection surveillance but is invasive and imperfectly captures diffuse myocardial injury. Cardiovascular magnetic resonance (CMR) offers noninvasive, multiparametric assessment of graft structure, function, tissue composition, and perfusion. We aimed to review current evidence supporting CMR in post-heart transplant surveillance and to evaluate the performance of serial CMR for acute cellular rejection in a prospective cohort. **Methods:** We performed a focused narrative review of the literature on CMR for detection of acute rejection, assessment of chronic allograft injury and prognosis, and evaluation of cardiac allograft vasculopathy and microvascular disease. In parallel, we conducted a prospective observational study of adult heart transplant recipients undergoing early post-transplant CMR (CMR1) and follow-up CMR (CMR2) with temporally matched EMB. Multiparametric CMR included cine imaging, native T1 and T2 mapping, extracellular volume fraction (ECV), and late gadolinium enhancement (LGE). Clinically significant acute cellular rejection was defined as ISHLT grade ≥ 2R. **Results:** Eighteen recipients were included (median 53 days to CMR1 and 192 days to CMR2). Baseline CMR parameters correlated with invasive hemodynamic and biomarkers. Two patients had biopsy-proven ≥2R rejection at follow-up. T2 values at CMR2 were significantly higher in rejection versus non-rejection patients (59.0 ± 1.4 ms vs. 51.1 ± 1.9 ms; *p* = 0.015), with greater LGE burden in rejection (*p* = 0.029). In longitudinal analyses, rejection was associated with divergent patterns of cardiac remodelling and tissue characterization, including increases in indexed ventricular volumes and T2 over time, whereas non-rejection patients demonstrated stable ventricular volumes and a decline in T2. **Conclusions:** Multiparametric CMR, anchored by T2 mapping, provides clinically meaningful, non-invasive information for acute rejection surveillance after heart transplantation and complements EMB within a personalized, risk-adapted follow-up framework. Establishing individualized baseline CMR phenotypes and monitoring longitudinal changes may support more personalized, less invasive graft surveillance strategies. Larger multicentre prospective studies are needed to define standardized implementation pathways.

## 1. Introduction

Heart transplantation remains the definitive therapy for selected patients with end-stage heart failure, but long-term outcomes continue to be limited by acute rejection, chronic allograft injury, and cardiac allograft vasculopathy (CAV). Early identification of graft injury is essential, as timely intervention can prevent irreversible myocardial damage and improve both graft and patient survival. Current surveillance strategies rely primarily on endomyocardial biopsy (EMB) for rejection detection and invasive coronary angiography for CAV assessment [1]. While these techniques remain the reference standards endorsed by international guidelines, they are invasive, resource-intensive, and incompletely capture the diffuse and heterogeneous myocardial and microvascular processes that characterize post-transplant graft pathology [1,2,3,4].

Cardiovascular magnetic resonance (CMR) has emerged as a comprehensive, non-invasive imaging modality for comprehensive graft assessment [2,3]. Through multiparametric evaluation of cardiac structure, function, tissue composition, and perfusion, CMR enables characterization of myocardial edema, interstitial remodelling, fibrosis, mechanical dysfunction, and ischemia within a single examination. Over the past decade, growing evidence has supported the diagnostic value of CMR—particularly T2 mapping, native T1 mapping, extracellular volume fraction (ECV), and myocardial strain—in the detection of acute cellular rejection (ACR) [5,6,7,8,9,10,11], as well as its prognostic relevance for chronic allograft injury and long-term outcomes [12,13]. Stress perfusion CMR further extends this capability to the functional assessment of CAV and microvascular disease, conditions that may be underestimated by anatomical imaging alone [13,14].

Despite this expanding body of evidence, the clinical integration of CMR into routine post-transplant surveillance remains heterogeneous. Importantly, no randomized controlled trials or prospective studies have established CMR as a direct replacement for EMB, and current recommendations continue to position CMR as a complementary rather than substitutive modality [3,4,15]. As a result, there is a critical need to clarify where CMR adds the greatest clinical value, how its findings should be interpreted longitudinally, and how it can be incorporated into personalized, risk-based surveillance strategies.

Among the spectrum of post-transplant complications, the detection and exclusion of clinically significant acute rejection represent the most time-sensitive and management-critical decision point. In this setting, CMR’s high sensitivity and negative predictive value raise the possibility that it may eventually inform future diagnostic pathways and support individualized clinical decision-making when used alongside EMB, biomarkers, and clinical assessment. While the diagnostic accuracy of individual CMR parameters is increasingly well-established, data illustrating their integration into real-world, longitudinal surveillance workflows remain limited. Therefore, this study combines a narrative review of current evidence with illustrative data from a small prospective observational cohort. Rather than seeking to establish new diagnostic thresholds, the purpose of this cohort is to provide a real-world demonstration of serial CMR application—specifically highlighting the value of a ‘baseline-plus-trajectory’ approach. By integrating established literature with this complementary clinical snapshot, we propose a personalized, guideline-aware framework for clinical implementation.

## 2. CMR in the Diagnosis of Acute Rejection After Heart Transplantation

CMR has emerged as a promising non-invasive modality for the assessment of acute rejection following heart transplantation, supported by a growing body of prospective studies, meta-analyses, and expert consensus statements. Its diagnostic value is primarily derived from multiparametric tissue characterization techniques, most notably T2 mapping, native T1 mapping, and ECV quantification, which directly reflect myocardial edema and interstitial expansion, the central pathophysiologic features of ACR [4,5,6,7,11].

Among available CMR techniques, T2 mapping has demonstrated the most consistent and robust diagnostic performance for ACR [8,11]. Meta-analytic data report pooled sensitivities and specificities of approximately 85–90%, with particularly high negative predictive value for clinically significant rejection [5]. Native T1 mapping and ECV provide complementary information by capturing diffuse myocardial injury and interstitial expansion, although their diagnostic specificity is lower [5,7]. Native T1 mapping has demonstrated sensitivities in the range of 80–85%, while ECV often shows very high sensitivity at the expense of reduced specificity, reflecting its responsiveness to multiple forms of myocardial remodelling beyond rejection alone [5].

In contrast, late gadolinium enhancement (LGE) has limited utility in the diagnosis of acute rejection. The diffuse, potentially reversible inflammatory changes that characterize ACR do not typically produce focal replacement fibrosis, resulting in modest sensitivities and specificities (approximately 50–60%) [5].

The diagnostic performance of CMR improves substantially when multiple parameters are interpreted in combination. Multiparametric approaches integrating T2 mapping with native T1 mapping or ECV have achieved sensitivities and negative predictive values approaching 100% for moderate-to-severe ACR (International Society for Heart and Lung Transplantation grade ≥2R) in selected cohorts, which reinforces the concept that CMR is particularly well suited as a rule-out tool [6,11].

Several diagnostic thresholds have been proposed, particularly for T2 mapping. At 1.5-Tesla scanners, optimal T2 cutoffs generally range between 54.75 and 59 ms, with reported sensitivities and specificities varying according to acquisition protocols and myocardial segment selection [5,8,11]. For example, a T2 cutoff at the basal level of approximately 57–58 ms has been associated with high specificity for biopsy-proven rejection [11]. ECV thresholds ≥ 32% have also demonstrated reasonable diagnostic accuracy, while native T1 cutoffs remain less standardized due to greater technical and inter-scanner variability [5,9,10,11].

Beyond isolated measurements, longitudinal assessment of CMR parameters provides additional diagnostic and clinical value. In fact, a significant limitation of the current evidence base is the heterogeneity of mapping protocols and scanner-specific variability. Consequently, many proposed diagnostic thresholds lack robust external validation. This variability highlights the difficulty of applying universal cutoffs in clinical practice, underscores the need for standardized multicenter protocols before absolute thresholds can be reliably adopted, and reinforces the value of monitoring an individual patient’s CMR trajectory over time. Mapping values typically decline over time after transplantation as ischemia–reperfusion injury and surgical inflammation resolve. Superimposed increases from an individual patient’s baseline may therefore signal acute rejection or other pathological processes. Serial monitoring of T2 and T1 mapping values allows detection of evolving myocardial injury, assessment of treatment response, and differentiation of rejection from transient, non-immunologic causes of myocardial edema [5,10].

This trajectory-based approach is particularly relevant early after transplantation, when mapping values may be elevated regardless of rejection, and when technical variability across scanners, sequences, and post-processing reduces the reliability of absolute cutoffs. In such settings, intra-patient changes over time provide a more robust and personalized indicator of pathological change than fixed population-based thresholds [5,10].

Beyond tissue characterization, CMR-derived myocardial strain, particularly feature-tracking strain obtained from standard cine images, adds a complementary functional dimension to rejection assessment. Recent prospective data demonstrate that global circumferential and radial strain accurately discriminate clinically significant ACR (≥2R), with areas under the curve of approximately 0.83–0.85 and very high negative predictive values (≈96–98%) [16]. Diagnostic performance improves further when strain is integrated with mapping parameters, with multiparametric models combining T2 and strain achieving AUCs exceeding 0.93 [9]. Strain parameters also improve following successful treatment of rejection, supporting their role in monitoring functional recovery [16]. Although standardization and multicentre validation are still evolving, strain enhances multiparametric CMR phenotyping by linking tissue abnormalities to clinically meaningful mechanical dysfunction.

Prospective studies have confirmed that global T1 and T2 values increase during episodes of acute rejection and correlate with other markers of graft injury, such as impaired myocardial strain and donor-derived cell-free DNA [10]. A randomized noninferiority trial comparing CMR to EMB has further demonstrated that CMR achieves sensitivities and negative predictive values exceeding 90% for the detection of ACR, reinforcing its clinical relevance as a non-invasive surveillance tool [17]. Reflecting this evidence, the American Heart Association identifies T2 mapping as the most promising CMR technique for detecting cellular rejection, while emphasizing that EMB remains the reference standard for diagnosis and classification [4].

A key limitation of CMR in the setting of acute rejection is its inability to reliably distinguish ACR from antibody-mediated rejection (AMR). Both entities produce overlapping myocardial phenotypes—edema, interstitial expansion, and fibrosis—leading to similarly elevated T2 and T1 values and increased ECV. As a result, CMR is sensitive for detecting rejection but lacks specificity for the underlying immunologic mechanism. Moreover, most published studies focus predominantly on cellular rejection, with limited data addressing AMR and no robust comparative analyses. Consequently, EMB remains indispensable for differentiating rejection subtypes through histopathologic and immunohistochemical evaluation [4,18].

## 3. CMR Markers of Chronic Allograft Injury and Long-Term Prognosis

As heart transplant recipients transition into long-term follow-up, morbidity and mortality are increasingly driven by chronic allograft injury rather than acute rejection. This process encompasses diffuse interstitial remodelling, replacement fibrosis, low-grade inflammation, microvascular dysfunction, and progressive functional impairment. These changes are often heterogeneous and spatially diffuse, limiting the sensitivity of endomyocardial biopsy and conventional imaging techniques. In this context, CMR provides a comprehensive, non-invasive platform for characterizing myocardial tissue composition and function and for stratifying long-term risk [12].

CMR tissue mapping parameters have demonstrated prognostic value in the long-term follow-up of heart transplant recipients, reflecting cumulative myocardial injury rather than acute inflammatory activity alone. In an adult prospective cohort undergoing CMR a median of 5.1–6.3 years after transplantation, elevated T2 (≥50.2 ms) emerged as the strongest independent predictor of adverse cardiac events, retaining significance even after hemodynamic adjustment. Conversely, the prognostic value of ECV fraction (>29%) was attenuated by hemodynamic factors, suggesting overlap with congestion, while native T1 lacked independent predictive value, reflecting stable interstitial remodelling [19]. Complementary evidence from a pediatric heart transplant cohort with a shorter follow-up after CMR demonstrated that native T1, ECV, and T2 were all independently associated with adverse outcomes, including cardiac hospitalization, transplantation, and cardiac death [20]. Collectively, these findings support a multiparametric approach where T2 consistently predicts risk, while the prognostic utility of T1 and ECV may be specific to pediatric or early-stage disease contexts.

LGE provides robust long-term prognostication by identifying irreversible myocardial fibrosis. Observational studies consistently link the presence and extent of LGE to adverse outcomes, independent of ejection fraction. These typically non-ischemic, patchy patterns reflect cumulative immune-mediated or microvascular injury and remain stable over time, signalling established damage rather than active disease. When combined with other markers, LGE identifies a high-risk phenotype characterized by significant cumulative graft injury [21,22].

Beyond tissue characterization, CMR-derived global longitudinal strain (GLS) offers sensitive detection of subclinical graft dysfunction. GLS independently predicts long-term mortality and adverse events, retaining prognostic value even after adjustment for ejection fraction and clinical history. Worsening strain serves as a functional integrator of diffuse pathology, with progressively impaired values signalling higher risk [23,24]. In addition, right ventricular longitudinal strain has emerged as an independent predictor of adverse outcomes and may add prognostic value beyond right ventricular ejection fraction, reflecting the importance of pulmonary vascular load and biventricular interaction in long-term graft performance [25,26].

## 4. CMR in Cardiac Allograft Vasculopathy and Microvascular Disease

CMR plays an important complementary role in the evaluation of CAV by enabling non-invasive assessment of both epicardial and microvascular disease, which are central to the pathophysiology of late graft failure. Unlike coronary angiography, which may underestimate early or diffuse disease, CMR interrogates the functional myocardial consequences of vascular pathology. In this context, stress perfusion CMR with quantitative myocardial perfusion reserve (MPR) has emerged as a key marker of CAV burden. Reduced MPR is independently associated with both epicardial and microvascular CAV and has demonstrated superior diagnostic performance compared with invasive angiography for detecting moderate-to-severe disease, particularly in diffuse or microvascular-predominant phenotypes [13,14].

Beyond epicardial involvement, CMR-derived MPR and strain-based parameters, including diastolic strain rate, enable detection of microvascular dysfunction before angiographic evidence of CAV. Impaired MPR and reduced diastolic strain rate correlate with histopathologic features of microvascular remodelling and reduced capillary density and are independently associated with adverse clinical outcomes and reduced event-free survival, even after adjustment for age, graft age, and angiographic CAV severity [14,27]. These findings underscore the value of CMR in identifying a high-risk ischemic phenotype that may otherwise remain occult with anatomical imaging alone.

In addition, LGE provides incremental prognostic information by detecting silent myocardial infarction and replacement fibrosis in transplant recipients with absent or mild angiographic CAV, reflecting the cumulative myocardial consequences of chronic ischemia and microvascular injury [28]. By integrating stress perfusion, strain, and tissue characterization, CMR moves beyond simple binary detection of CAV, allowing for a more nuanced assessment of disease severity and myocardial risk. Accordingly, contemporary heart transplant guidelines recognize CMR as a complementary tool for long-term allograft surveillance and CAV screening, particularly within a personalized, risk-based follow-up strategy [3].

## 5. Methods

### 5.1. Study Design and Rationale

We conducted a prospective observational cohort study to evaluate the role of serial CMR in the surveillance of acute cellular rejection. To clarify our clinical focus, the objectives of this cohort study were defined as follows:

• Primary Objective: To evaluate the longitudinal changes in mapping parameters and ventricular remodeling after transplantation, and to determine how these trajectories differ according to the presence of biopsy-proven acute cellular rejection.
• Secondary Objectives: To evaluate the cross-sectional discrimination of concurrent rejection using CMR parameters (specifically T2 mapping) at the time of follow-up biopsy, and to explore the baseline physiologic correlation of CMR indices with invasive hemodynamics and circulating biomarkers.

By structuring the analysis this way, we aimed to contextualize cross-sectional CMR findings within the broader longitudinal evolution of the allograft.

### 5.2. Study Population

We prospectively enrolled consecutive adult heart transplant recipients followed at Hospital de Santa Marta - Unidade Local de Saúde São José tertiary transplant centre between January 2024 and June 2025. Eligible patients underwent an early post-transplant CMR (CMR1) and a follow-up CMR (CMR2) as part of a structured surveillance protocol.

During the study period, a total of 22 consecutive adult heart transplant recipients were evaluated for inclusion. Four patients were excluded prior to the baseline scan: one declined to participate due to claustrophobia, one was clinically unstable and unable to undergo CMR, one had a standard contraindication to CMR, and one died. The remaining 18 patients successfully completed the imaging protocol and were included in the final analysis. As this was an exploratory feasibility study intended to illustrate real-world longitudinal CMR tracking, no formal *a priori* sample size calculation was performed. The sample size was dictated entirely by the consecutive volume of eligible transplantations and the feasibility of performing paired CMR and EMB during the pre-defined study period.

A total of 18 heart transplant recipients were included in the final analysis. The median time from transplantation to CMR1 was 53 days, and to CMR2 was 192 days. EMB was performed in close temporal proximity to each CMR examination according to institutional practice, with the exact time intervals between procedures calculated and reported.

### 5.3. Endomyocardial Biopsy and Definition of Rejection

Endomyocardial biopsy was used as the reference standard for rejection assessment. Biopsies were graded according to the International Society for Heart and Lung Transplantation (ISHLT) classification. Clinically significant acute cellular rejection was defined as grade ≥2R.

### 5.4. CMR Acquisition Protocol

All CMR examinations were performed on the same 1.5-Tesla scanner (Magnetom Sola, Siemens Healthineers, Erlangen, Germany), using a standardized protocol to minimize inter-scan variability. The protocol included:

• Cine steady-state free precession sequences for assessment of biventricular volumes and function.
• Native T1 mapping (acquired using a 5-3-3 Modified Look-Locker Inversion recovery [MOLLI] sequence).
• T2 mapping (acquired using a T2-prepared bSSFP sequence).
• Post-contrast T1 mapping for calculation of extracellular volume fraction (ECV) acquired following the administration of gadolinium-based contrast.
• Late gadolinium enhancement (LGE) imaging for detection of focal myocardial fibrosis or infarction.

Mapping values were assessed using a standardized segmentation approach, and global values were derived by averaging segmental measurements. LGE was categorized as ischemic or non-ischemic based on pattern and distribution.

### 5.5. CMR Analysis

Left and right ventricular end-diastolic and end-systolic volumes were indexed to body surface area, and ejection fractions were calculated using standard methods. Mapping parameters (native T1, T2, and ECV) were analysed globally, and changes between CMR1 and CMR2 were calculated to assess temporal trajectories.

LGE presence, extent, and pattern were assessed qualitatively and semi-quantitatively. All image post-processing and analyses were performed using dedicated, commercially available software (cvi42, Circle Cardiovascular Imaging, v6.0.3). To ensure measurement reproducibility and to mitigate single-observer bias—particularly regarding the semi-quantitative grading of LGE burden—all CMR findings and segmentations were evaluated and validated by a second, independent, experienced CMR physician. Both observers were blinded to the temporally matched endomyocardial biopsy results. Any discrepancies in LGE grading or mapping contours between the primary reader and the second validator were resolved by consensus.

### 5.6. Hemodynamic Assessment

Right-heart catheterization data obtained in proximity to CMR were collected when available. Hemodynamic parameters were used to explore coherence between invasive measurements and CMR-derived functional indices.

### 5.7. Outcomes

The primary outcome was the presence of biopsy-proven moderate-to-severe acute cellular rejection (≥2R) at the time of CMR2. Secondary exploratory outcomes included differences in ventricular remodelling, mapping trajectories, and LGE characteristics between patients with and without rejection.

### 5.8. Statistical Analysis

Continuous variables are presented as mean ± standard deviation or median [interquartile range], depending on the distribution of the data. The assumption of normality was evaluated using the Shapiro–Wilk test and visual inspection of histograms. Given the small sample size, non-parametric tests were primarily utilized. Comparisons of continuous variables between patients with and without rejection were performed using the Mann-Whitney U test (exact *p*-values). Within-patient longitudinal changes between CMR1 and CMR2 were assessed using the Wilcoxon signed-rank test. Correlations between CMR parameters and invasive hemodynamics or biomarkers were assessed using Pearson’s correlation coefficient for normally distributed data, or Spearman’s rank correlation for non-normally distributed data.

Receiver operating characteristic (ROC) curve analysis was performed exploratorily to calculate the area under the curve (AUC) for T2 mapping at follow-up (CMR2). We emphasize that due to the very low number of outcome events (*n* = 2), this ROC analysis was not prespecified to derive new, robust diagnostic thresholds. Rather, it was utilized strictly as a descriptive statistical tool to quantify the degree of cross-sectional discrimination observed within this specific, small cohort. All tests were two-sided, and a *p*-value < 0.05 was considered statistically significant.

## 6. Results

Eighteen heart transplant recipients were included. Baseline clinical, CMR, and invasive hemodynamic characteristics are summarized in Table 1. The median ischemic time was 232 min, and the median time from transplantation to the first CMR examination was 53 days. All patients were maintained on standard triple immunosuppressive therapy comprising tacrolimus, mycophenolate mofetil, and prednisone. The median time interval between CMR1 and the matched baseline EMB was −1.5 days (IQR [−8.0, 2.5]), and the interval between CMR2 and the follow-up EMB was 2.0 days (IQR [−6.5, 5.0]). At baseline (Table 1), left and right ventricular volumes and systolic function were normal. Native T1 and T2 values were mildly elevated, consistent with early post-transplant myocardial edema and inflammatory remodelling. Late gadolinium enhancement was present in 8 patients (44%), including ischemic-pattern LGE in 3 (17%); among LGE-positive patients, extent was classified as low (1–2 segments) in 5/8, moderate (3–4 segments) in 2/8, and severe (≥5 segments) in 1/8. Invasive hemodynamics demonstrated preserved cardiac output with low-to-moderate filling pressures, low pulmonary pressures, and preserved right ventricular–pulmonary vascular coupling at the time of early CMR assessment.

Exploratory correlation analyses at baseline assessed the relationship between CMR-derived functional indices, invasive hemodynamics, and circulating biomarkers. As expected, LV stroke volume index correlated positively with catheterization-derived cardiac index (r = 0.63, *p* = 0.005), and higher pulmonary vascular resistance was associated with lower right ventricular ejection fraction (r = −0.47, *p* = 0.047), supporting physiologic concordance between non-invasive and invasive measures. In biomarker analyses, higher troponin concentrations were associated with lower biventricular forward volumes, including LV stroke volume index (ρ = −0.67, *p* = 0.002), RV end-diastolic volume index (ρ = −0.59, *p* = 0.010), and RV stroke volume index (ρ = −0.58, *p* = 0.011). No other statistically significant correlations were observed.

In the overall cohort, the median interval from heart transplantation to CMR2 was 192 days. Among patients without rejection at follow-up, ventricular volumes and ejection fraction remained stable between CMR1 and CMR2 (all *p* > 0.5). Mapping parameters showed a trend toward T1 reduction (ΔT1 −31.9 ms, *p* = 0.064), while T2 decreased significantly over time (ΔT2 −2.33 ms, *p* = 0.029). ECV did not change significantly over time (*p* = 0.164).

**Figure 1:**
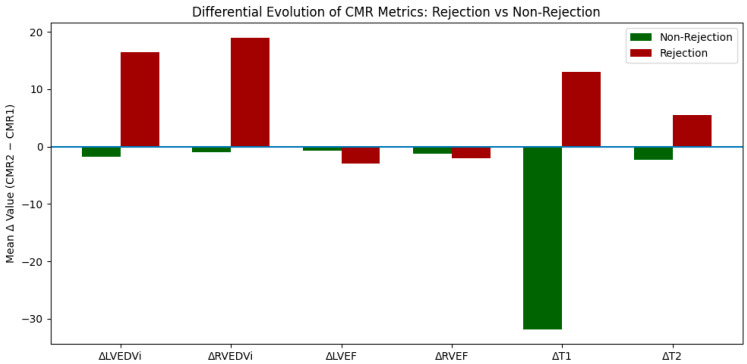
Differential evolution of CMR parameters between CMR1 and CMR2 according to rejection status. Bars represent mean within-patient changes (Δ = CMR2 − CMR1) in LVEDVi, LVESVi, LVEF, RVEDVi, RVESVi, RVEF, native T1, and T2.

Two patients had biopsy-proven clinically significant acute cellular rejection (≥2R) at follow-up. No episodes of antibody-mediated rejection were identified during the study period. Compared with non-rejection patients, those with rejection demonstrated higher T2 values at CMR2 (59.0 ± 1.4 ms vs. 51.1 ± 1.9 ms; *p* = 0.015) and more extensive LGE burden at CMR2 (*p* = 0.029). Importantly, longitudinal trajectories differed: rejection patients exhibited increases in indexed ventricular volumes, with ΔLVEDVi +16.5 vs. −1.7 mL/m^2^ in non-rejection (*p* = 0.088) and ΔRVEDVi +19.0 vs. −0.9 mL/m^2^ (*p* = 0.015). Mapping trajectories also diverged, with an increase in T2 in rejection patients (ΔT2 +5.5 ms vs. −2.33 ms, *p* = 0.029), while T1 showed opposite directional changes (+13.0 vs. −31.9 ms), although this did not reach statistical significance (*p* = 0.235). These cross-sectional differences at follow-up and divergent longitudinal trajectories are summarized in Table 2, and the contrasting direction and magnitude of CMR changes between rejection and non-rejection patients are visually depicted in Figure 1.

Additionally, T2 values at follow-up (CMR2) demonstrated excellent discrimination for biopsy-proven clinically significant acute cellular rejection (≥2R), with an AUC of 1.00 in this cohort. Notably, there was no overlap in T2 values between rejection and non-rejection patients. We emphasize that this perfect discrimination and absence of overlap reflects the extremely small number of events rather than a reproducible diagnostic threshold, and should be interpreted strictly as a descriptive trend.

## 7. Discussion: Clinical Role of CMR in Personalized Post-Transplant Surveillance

In the absence of universally accepted CMR-based surveillance protocols, current evidence supports a complementary and personalized role for CMR in the follow-up of heart transplant recipients. Importantly, there is currently no evidence from randomized controlled trials or prospective studies supporting CMR as a direct replacement for EMB in post-transplant surveillance. Accordingly, CMR should not be viewed as a substitute for EMB, but rather as a non-invasive adjunct that enhances diagnostic confidence and refines risk stratification, in line with existing guideline recommendations.

From a clinical perspective, the greatest immediate utility of CMR lies in the detection and exclusion of clinically significant ACR. As established in prior large prospective studies and meta-analyses, multiparametric tissue characterization—particularly T2 mapping, supported by native T1 mapping, ECV, and myocardial strain—provides high sensitivity and negative predictive value for moderate-to-severe rejection. These metrics must be contextualized carefully; because predictive values depend strongly on disease prevalence, the high negative predictive value reported in the literature is mathematically driven, in part, by the relatively low baseline prevalence of clinically significant acute rejection in contemporary transplant cohorts. This reinforces CMR’s established utility primarily as a non-invasive ‘rule-out’ test. Nonetheless, our prospective cohort data are concordant with this prior literature: patients with biopsy-proven clinically significant ACR (≥2R) demonstrated significantly higher T2 values at follow-up CMR compared with non-rejection patients (59.0 ± 1.4 ms vs. 51.1 ± 1.9 ms; *p* = 0.015). In this dataset, T2 mapping showed complete separation of clinically significant rejection at follow-up, reinforcing the central role of myocardial edema as the dominant imaging phenotype of acute cellular rejection. While the apparent perfect separation observed in this small cohort should be interpreted cautiously, these findings support the clinical value of T2 mapping as a highly sensitive rule-out tool and as a trigger for closer monitoring or invasive confirmation in selected clinical contexts.

Beyond mapping, our results suggest that clinically significant rejection may be accompanied by a broader myocardial injury phenotype. Patients with ≥2R exhibited a greater LGE burden at follow-up, consistent with more extensive focal myocardial injury in the setting of rejection. Although LGE is not considered a primary diagnostic marker of acute rejection, its extent may provide complementary information and contextualize mapping abnormalities within a broader graft injury profile. However, because LGE is not specific for acute rejection, this finding is difficult to interpret in isolation; it may reflect other concurrent processes such as perioperative injury, evolving fibrosis, or cumulative myocardial vulnerability.

Importantly, while our cohort was not designed to establish definitive trajectory-based thresholds, we observed divergent patterns of ventricular remodelling and tissue evolution between rejection and non-rejection patients, with volume expansion and increasing T2 in those with rejection and relative stability or normalization in those without. These findings are consistent with the concept that clinically meaningful rejection manifests not only as an isolated tissue abnormality but as an integrated myocardial phenotype encompassing edema, injury burden, and early remodelling. In contrast, the stability of ventricular function and the decline in T2 observed in non-rejection patients likely reflect resolution of early post-transplant inflammatory changes.

As detailed in the narrative review portion of this manuscript, multiparametric CMR enables phenotyping of chronic myocardial injury beyond acute rejection by integrating markers of diffuse interstitial remodelling (native T1, ECV), persistent edema or inflammation (T2), focal replacement fibrosis (LGE), and subclinical mechanical dysfunction (strain). These complementary parameters capture the cumulative burden of graft injury and provide prognostically meaningful information when interpreted together. Importantly, these myocardial phenotypes likely reflect both immune-mediated injury and downstream consequences of occult microvascular dysfunction or early cardiac allograft vasculopathy (CAV), underscoring the need for integrated interpretation rather than isolated parameter assessment. While we emphasize that these chronic endpoints and CAV were not specifically tested in our short-term prospective cohort, the reviewed literature highlights the broader value of CMR for long-term risk stratification, chronic allograft injury assessment, and functional evaluation of CAV and microvascular disease.

In routine follow-up, these exploratory findings suggest how CMR might eventually be incorporated within a personalized surveillance strategy, particularly in clinically stable or lower-risk patients. Early post-transplant CMR can establish individualized baseline tissue characteristics, while subsequent examinations—performed at predefined intervals or triggered by clinical or biomarker changes—allow detection of pathological deviations from baseline. This baseline-plus-trajectory approach may mitigate inter-individual variability and scanner-dependent effects, allowing CMR findings to be interpreted in a patient-specific longitudinal context rather than against fixed population thresholds alone. When integrated with clinical assessment, biomarkers, echocardiography, and invasive testing, CMR provides a comprehensive, patient-centred evaluation of graft health.

Within this framework, CMR functions primarily as a decision-support, risk-stratification, and phenotyping tool rather than a definitive diagnostic replacement for established invasive strategies. Given the central importance of acute rejection detection to clinical management—and the strong diagnostic signal demonstrated by T2 mapping in both prior literature and our cohort—prospective evaluation of CMR-centred, risk-adapted surveillance pathways represents a critical next step toward a more personalized, less invasive model of post-heart transplant care.

## 8. Limitations and Future Directions

This study has several limitations. First, the cohort was small and single-centre, limiting statistical power and generalizability; in particular, the number of patients with clinically significant acute cellular rejection was low (*n* = 2)—consequently, the comparative statistical analyses and estimated parameters presented here carry a high degree of uncertainty and must be interpreted strictly as exploratory and hypothesis-generating. Furthermore, we did not adjust for multiple comparisons; therefore, nominally significant findings should be interpreted with caution, as the risk of Type I errors is increased. Second, although all CMR studies were acquired using a standardized protocol on the same scanner, absolute mapping values are influenced by sequence- and vendor-specific factors, which may limit portability of thresholds across centres. Third, while longitudinal changes were analysed, the study was not powered to define trajectory-based cutoffs or to quantify the incremental value of serial CMR over single time-point assessment.

No cases of antibody-mediated rejection occurred in this cohort; therefore, CMR performance for AMR and its ability to discriminate rejection phenotypes could not be assessed. Additionally, long-term clinical outcomes beyond acute rejection were not evaluated, and correlations with invasive hemodynamics and biomarkers should be interpreted as exploratory and hypothesis-generating. Similarly, ROC analyses—while supportive of strong discrimination of ≥2R rejection by T2 at follow-up—were based on a very small number of rejection events. This complete separation in a small sample introduces a substantial risk of optimism bias, and these metrics likely overestimate true diagnostic performance. Future work should validate these findings in larger multicenter prospective cohorts and evaluate how CMR can be integrated into safe, risk-adapted surveillance pathways.

Finally, the literature review component of this manuscript was conducted as a focused narrative review rather than a formal systematic review; consequently, it lacks a structured search methodology and may be subject to literature selection bias.

## 9. Conclusions

In this small, exploratory prospective cohort of heart transplant recipients undergoing serial cardiovascular magnetic resonance imaging, T2 mapping demonstrated strong discriminatory ability for clinically significant acute cellular rejection, supporting its role as a sensitive, noninvasive marker of graft inflammation, rather than establishing a robust diagnostic threshold. Beyond isolated tissue characterization, CMR provided integrated insights into myocardial injury and remodeling patterns that differ according to rejection status.

Taken together with the existing literature, these exploratory findings support a complementary, personalized role for CMR in post-heart transplant surveillance. Rather than replacing endomyocardial biopsy, CMR may enhance diagnostic confidence, refine risk stratification, and enable patient-specific longitudinal assessment when incorporated into multimodal surveillance strategies. Establishing individualized baseline CMR phenotypes and monitoring trajectories over time may represent a pragmatic step toward more personalized, less invasive graft monitoring in selected clinical contexts. Ultimately, larger prospective multicenter studies with adequate event numbers will be necessary before CMR parameters can be considered reliable, standalone tools for routine clinical rejection surveillance.

## Figures and Tables

**Table 1:** Baseline characteristics, CMR, and invasive hemodynamics at first post-transplant evaluation (CMR1). Values are presented as mean ± SD or median [interquartile range], as appropriate. Ischemic time refers to donor organ ischemic time. LGE extent was graded semi-quantitatively using an ordinal score (light: 1–2 segments; moderate: 3–4 segments; severe: ≥5 segments). Hemodynamic variables were obtained by right-heart catheterization. Abbreviations: CMR, cardiovascular magnetic resonance; LVEDVi/LVESVi, left ventricular end-diastolic/end-systolic volume index; LVEF, left ventricular ejection fraction; RVEDVi/RVESVi, right ventricular end-diastolic/end-systolic volume index; RVEF, right ventricular ejection fraction; T1/T2, native myocardial relaxation times; ECV, extracellular volume fraction; LGE, late gadolinium enhancement; PAPi, pulmonary artery pulsatility index.

Baseline Characteristics	
**Age**, years	51.0 ± 17.2
**Male sex**, n (%)	9 (50%)
**Ischemic time**, min	232 [155–272]
**Time from transplant to CMR1**, days	53 [41–71]
**Baseline CMR (CMR1)**	
**LVEDVi**, ml/m^2^	70.2 ± 13.8
**LVESVi**, ml/m^2^	28.3 ± 9.2
**LVEF**, %	61.4 ± 7.4
**RVEDVi**, ml/m^2^	71.5 ± 17.1
**RVESVi**, ml/m^2^	28.8 ± 10.1
**RVEF**, %	60.3 ± 6.9
**Native T1**, ms	1094.7 ± 61.2
**T2**, ms	53.3 ± 4.3
**ECV**, %	29.9 ± 3.6
**LGE present**, n (%)	8 (44%)
**Ischemic LGE**, n (%)	3 (17%)
**LGE extent (semi-quantitative)**, n (%)	
Light (score 1–2)	5 (28%)
Moderate (score 3–4)	2 (11%)
Severe (score ≥5)	1 (6%)
**Baseline invasive hemodynamics**	
**Right atrial pressure**, mmHg	1.3 [1.1–1.6]
**Mean pulmonary artery pressure**, mmHg	17.5 [15.3–18.8]
**Pulmonary capillary wedge pressure**, mmHg	9.0 [6.3–11.0]
**Cardiac index**, L/min/m^2^	3.34 [3.11–3.70]
**Cardiac output**, L/min	6.0 [4.94–6.76]
**Pulmonary artery pulsatility index** (PAPi)	2.8 [2.23–3.56]

**Table 2:** Longitudinal CMR changes according to acute cellular rejection at follow-up biopsy (≥2R). Values are mean ± SD. Δ indicates mean change from CMR1 to CMR2. Within-group p-values refer to differences between CMR2 and CMR1 in non-rejection patients (Wilcoxon signed-rank). Between-group p-values compare Δ trajectories between non-rejection and rejection patients (Mann–Whitney U; exact *p*).

Parameter	No Rejection (n = 16) CMR1	No Rejection (n = 16) CMR2	Δ No Rejection	*p*	Rejection (n = 2) CMR1	Rejection (n = 2) CMR2	Δ Rejection	*p* (Δ Between Groups)
**LVEDVi** (ml/m^2^)	70.3 ± 13.6	68.6 ± 8.8	−1.7	0.530	59.5 ± 5.0	76.0 ± 15.6	+16.5	0.088
**LVESVi** (ml/m^2^)	29.3 ± 8.9	27.9 ± 7.4	−1.3	0.392	17.5 ± 2.1	24.5 ± 9.2	+9.5	0.029
**LVEF** (%)	60.3 ± 7.2	59.5 ± 7.9	−0.7	0.624	71.0 ± 1.4	68.0 ± 5.7	−3.0	0.529
**RVEDVi** (ml/m^2^)	70.9 ± 13.8	70.0 ± 12.5	−0.9	0.909	55.5 ± 13.4	74.5 ± 16.3	+19.0	**0.015**
**RVESVi** (ml/m^2^)	40.7 ± 9.0	40.1 ± 6.9	−0.6	0.753	40.5 ± 5.0	50.5 ± 7.8	+10.0	0.059
**RVEF** (%)	58.9 ± 4.6	57.7 ± 5.9	−1.2	0.648	74.0 ± 8.5	72.0 ± 9.9	−2.0	0.618
**Native T1** (ms)	1094 ± 63	1063 ± 41	−31.9	0.064	1132 ± 17.7	1144 ± 0.7	+13.0	0.235
**T2** (ms)	53.4 ± 4.5	51.1 ± 1.9	−2.3	**0.029**	53.5 ± 5.0	59.0 ± 1.4	+5.5	**0.029**

## Data Availability

The data presented in this study are available upon request from the corresponding author. They are not publicly available due to privacy and data protection regulations (Regulamento Geral sobre a Proteção de Dados—[RGPD] (EU)2016/679).
